# Nivolumab in chemotherapy-resistant cervical cancer: report of a vulvitis as a novel immune-related adverse event and molecular analysis of a persistent complete response

**DOI:** 10.1186/s40425-019-0742-6

**Published:** 2019-10-31

**Authors:** Florence Baettig, Tatjana Vlajnic, Marcus Vetter, Katharina Glatz, Jürgen Hench, Stephan Frank, Michel Bihl, Roberto Lopez, Michael Dobbie, Viola Heinzelmann-Schwarz, Céline Montavon

**Affiliations:** 1 Department of Gynaecology and Obstetrics, Hôpital du Jura, Delémont, Switzerland; 2 Institute of Pathology, University Hospital Basel, Basel, Switzerland; 3 Gynaecological Cancer Centre, University Hospital Basel and University of Basel, Basel, Switzerland; 4 Department of Oncology, Hôpital du Jura, Delémont, Switzerland; 5 Hospital for Women, Gynaecological Oncology, University Hospital Basel, Spitalstrasse 21, 4031 Basel, Switzerland

**Keywords:** Immunotherapy, Nivolumab, Immune-related adverse event, Vulvitis, Cervical cancer therapy, Chemotherapy, DNA methylation profiling, Human papillomavirus, Tumour mutational burden, Copy number profile

## Abstract

**Background:**

Treatment options for advanced cervical cancer are limited and patients experiencing recurrence after first-line cisplatin-based chemotherapy and bevacizumab have a poor prognosis. A recent phase II study in advanced cervical cancer has demonstrated a disease control rate of 68.4% with the immune checkpoint inhibitor nivolumab. By blocking immune checkpoints, immunotherapy puts the immune system into a state of hyper-activation that can cause immune-related adverse events.

We present the clinical, pathological and molecular data of a patient with metastatic cervical cancer and progressive disease after second-line therapy. We report on the therapeutic response under third-line immunotherapy with nivolumab, the immune-related adverse events (IRAE), and their successful management.

**Case presentation:**

We report the case of a 62-year-old woman who was diagnosed with advanced squamous cell carcinoma of the cervix with paraaortic lymph node metastases. After an initial combined radio-chemotherapy with cisplatin, she developed local and nodal (supraclavicular) recurrence. Second-line chemotherapy with 6 cycles of carboplatin, paclitaxel, and bevacizumab resulted in a partial response for 6 months. Checkpoint inhibition with nivolumab was started due to progression, leading to persistent complete remission.

Immunotherapy was well tolerated for 8 months until the patient presented with an immune-related isolated vulvitis, which was successfully managed with topical corticosteroids.

**Conclusions:**

The persistent complete response after third-line treatment for relapsed chemotherapy-resistant cervical cancer presented in this case highlights the potential of immunotherapy for patients with advanced cervical cancer impressively.

To our knowledge, this is the first report of an isolated immune-related vulvitis under nivolumab. This adverse event might be underdiagnosed and mistreated, however, it is of importance due to its impact on quality of life, sexual wellbeing and compliance of patients. Successful IRAE management may enable prolonged immune checkpoint inhibitor therapy. In the future, routine molecular tumour profiling is likely to aid in the stratification of cervical cancer patients for immunotherapy. Here, we provide the methylome data of a case with complete response.

## Background

Immunotherapy with immune checkpoint inhibitors has emerged as a novel option for many patients with advanced cancers whom previously had limited treatment options and experienced poor outcomes. While clinical studies have demonstrated survival benefits and durable responses in various cancer entities, immunotherapy of gynaecological cancers is still in relative infancy. In the United States, pembrolizumab, an antibody against programmed cell death protein 1 (PD-1), is approved for advanced endometrial cancers with high levels of microsatellite instability (MSI-high) and for recurrent or progressive metastatic cervical cancer positive for programmed death-ligand 1 (PD-L1), suggesting that these parameters could serve as predictive biomarkers.

Under physiological conditions, immune checkpoints play a crucial role in preventing autoimmunity [1]. Through the expression of PD-L1, cancer cells modulate the immune checkpoint to downregulate T cells, thereby protecting themselves from immune attack [2]. Immune checkpoint inhibitors reduce the interaction between cancer cells and T cells so that re-activated lymphocytes can destroy their malignant targets. Antibodies against PD-1 such as nivolumab have recently been approved for the treatment of different tumour types, particularly melanoma and non-small cell lung cancer [2].

More than 95% of cervical cancers are caused by a human papillomavirus (HPV) infection. Despite Papanicolaou testing, HPV screening, and prophylactic HPV vaccination, cervical cancer remains the fourth most common cause of death by cancer in women worldwide. The 5-year overall survival (OS) of recurrent or metastatic cervical cancer is poor (around 15%), mainly due to limited treatment options.

For the last 20 years, the standard of care for patients with recurrent or metastatic cervical cancer has been cisplatin-based chemotherapy combined with paclitaxel. However, despite the addition of other agents such as vinorelbine, gemcitabine or topotecan [3], most patients deteriorated rapidly, developing platinum resistance at recurrence [4]. Adding bevacizumab, an anti-vascular endothelial growth factor antibody, to chemotherapy became the standard of care since the Gynecologic Oncology Group 240 trial demonstrated a survival benefit of almost 4 months [5]. Recent evidence indicates the potential usefulness of immune checkpoint inhibitors in cervical cancer [4] with objective response rates (ORR) in recurrent and/or advanced cervical cancer ranging from 12.2 to 26% [6]. However, it remains challenging to select patients with a potential therapeutic response and to anticipate the magnitude of response. Predictive markers of therapeutic response to immunotherapy are still unclear. Increased PD-L1 expression by tumour and immune cells as well as elevated tumour mutational burden (TMB; mutations per tumour genome coding region) have been associated with an increased likelihood of response [7]. High-TMB tumours tend to have more neoantigens serving as potential targets for the immune system [8]. Furthermore, patients with microsatellite-unstable (MSI-high) tumours seem to qualify for immunotherapy regardless of tumour type [9]. Last, immune responsiveness signatures based on methylation profiles, as already identified in lung cancer, are rapidly moving into clinical focus [10].

Whereas immune checkpoints are crucial in maintaining self-tolerance, their inhibition by immunotherapy puts the immune system into a hyperactive state that can cause immune-related adverse events (IRAE). IRAEs are frequent and occur in up to 70% of patients treated with anti-PD1/PD-L1 antibodies. Typically, IRAEs occur within weeks to a few months after treatment initiation, yet, reports on delayed IRAEs one year after treatment discontinuation have also been recorded [11]. Any organs can be affected by an IRAE, which can, in some cases, be life-threatening or even lethal [11].

We report a patient with persistent complete response after third-line treatment with Nivolumab for relapsed chemotherapy-resistant, high-TMB cervical cancer, which underlines the potential of immunotherapy. In addition, we describe vulvitis as an isolated IRAE under nivolumab, which was successfully treated with topical corticosteroids.

## Case presentation

A 62-year-old woman was diagnosed with squamous cell carcinoma of the cervix uteri FIGO Stage IIIC2(r) (according to FIGO 2018). Computed tomography (CT) at first diagnosis showed a large cervical mass of 9 cm, enlarged iliac and paraaortic lymph nodes, and right hydronephrosis. Prior personal and family history were negative. First-line treatment included combined radio-chemotherapy with 6 cycles of cisplatin 40 mg/m^2^ weekly and lymph node irradiation, which was followed by intra-cervical brachytherapy with Iridium-192 resulting in a partial response. Three months later, progressive disease with a new palpable mass in a left supraclavicular lymph node and suspicion of pulmonary metastases (small nodules of 6 mm, not amenable to biopsy) was noted. A mixed response of the pelvic nodal metastases as well as a progression of the cervical mass was documented. After radiotherapy of the left supraclavicular lymph node metastasis and second-line chemotherapy with 6 cycles of carboplatin/AUC6, paclitaxel 175 mg/m^2^, and bevacizumab 15 mg/kg, partial response (regression of all lesions without complete resolution) for 6 months was achieved. However, bevacizumab had to be interrupted due to rectorrhagia and ensuing anaemia**.** Due to progressive disease with new retrocrural and paraesophageal lymph node metastases, local radiotherapy was applied and a third-line systemic treatment with nivolumab (3 mg/kg q2w for 17 months, then according to Checkmate-358 with 240 mg q2w) was initiated leading to complete remission as verified by positron emission tomography-computed tomography (PET-CT) at 12 months after immunotherapy initiation. Complete remission was also documented after 22 months of treatment.

**Fig. 1:**
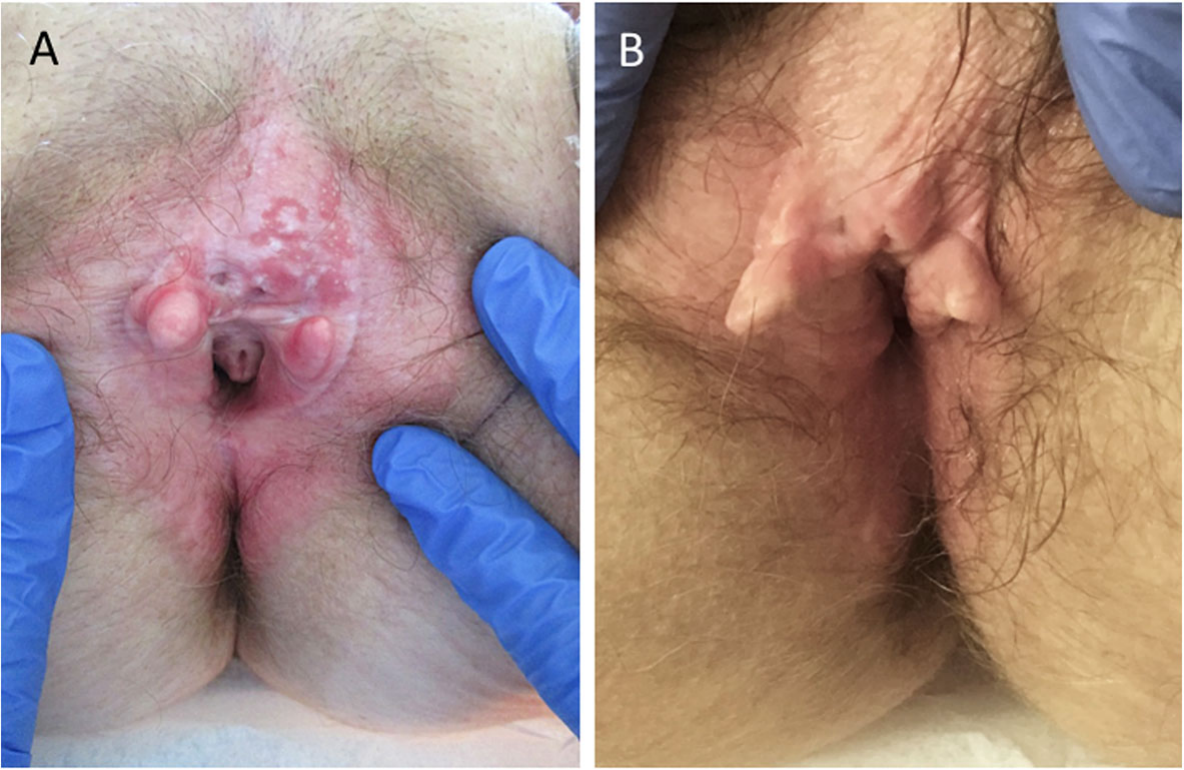
Clinical appearance and successful treatment of the IRAE. **a** Ulcerative vulvitis six months after initial IRAE symptoms; biopsy shown in Fig. 2 was taken. **b** Successful management of IRAE with topical corticosteroids

**Fig. 2:**
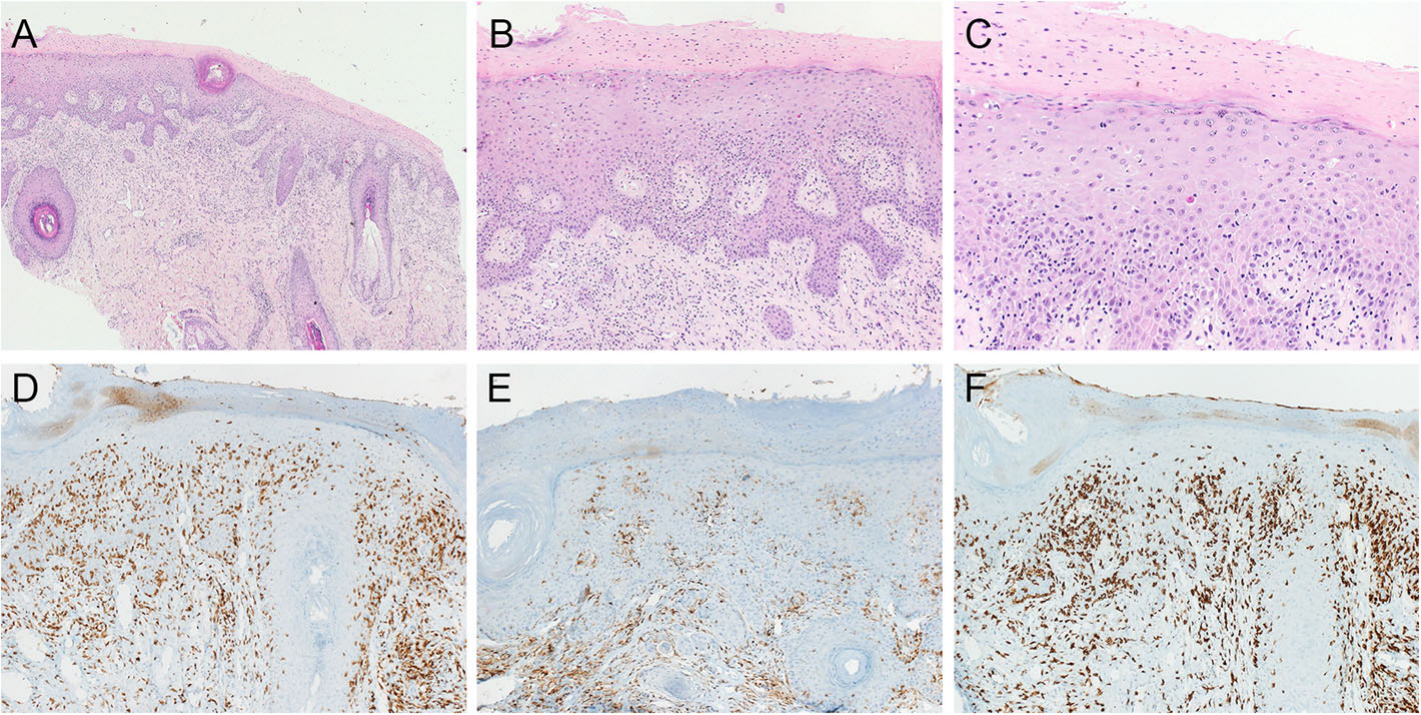
**a-c** Biopsy of the vulva with lichenoid interface dermatitis: Squamous epithelium with hyper- and parakeratosis, irregular acanthosis, and dense band-like and perivascular lymphocytic infiltrate along the dermo-epidermal junction as well as intraepithelial lymphocytic infiltrate. Scattered necrotic keratinocytes (original magnification **a** 40x, **b** 100x, **c**, 200x). **e-f** Immunohistochemistry for T cell markers CD3, CD4, and CD8: Intraepithelial T cells predominantly express CD8 (original magnification **e**, **f** 100x)

The immunotherapy was well tolerated over the course of 8 months, then the patient presented with a well-delimited inflammation of the vulva and perianal region with ulceration and epidermolysis (Fig. 1a). A bacterial, fungal, or viral infection was excluded by microbiological analyses. Histology of a punch biopsy revealed a lichenoid interface dermatitis with a pronounced lymphocytic infiltrate along the dermo-epidermal junction as well as intraepithelial. Immunohistochemically, most of lymphocytes were CD8-positive T cells intermixed with CD4-positive T cells (Fig. 2), consistent with an immunotherapy-related vulvitis. This IRAE was successfully treated by topical corticosteroid (clobetasol propionate 0.05%, 1-2x daily; Fig. 1b).

**Fig. 3:**
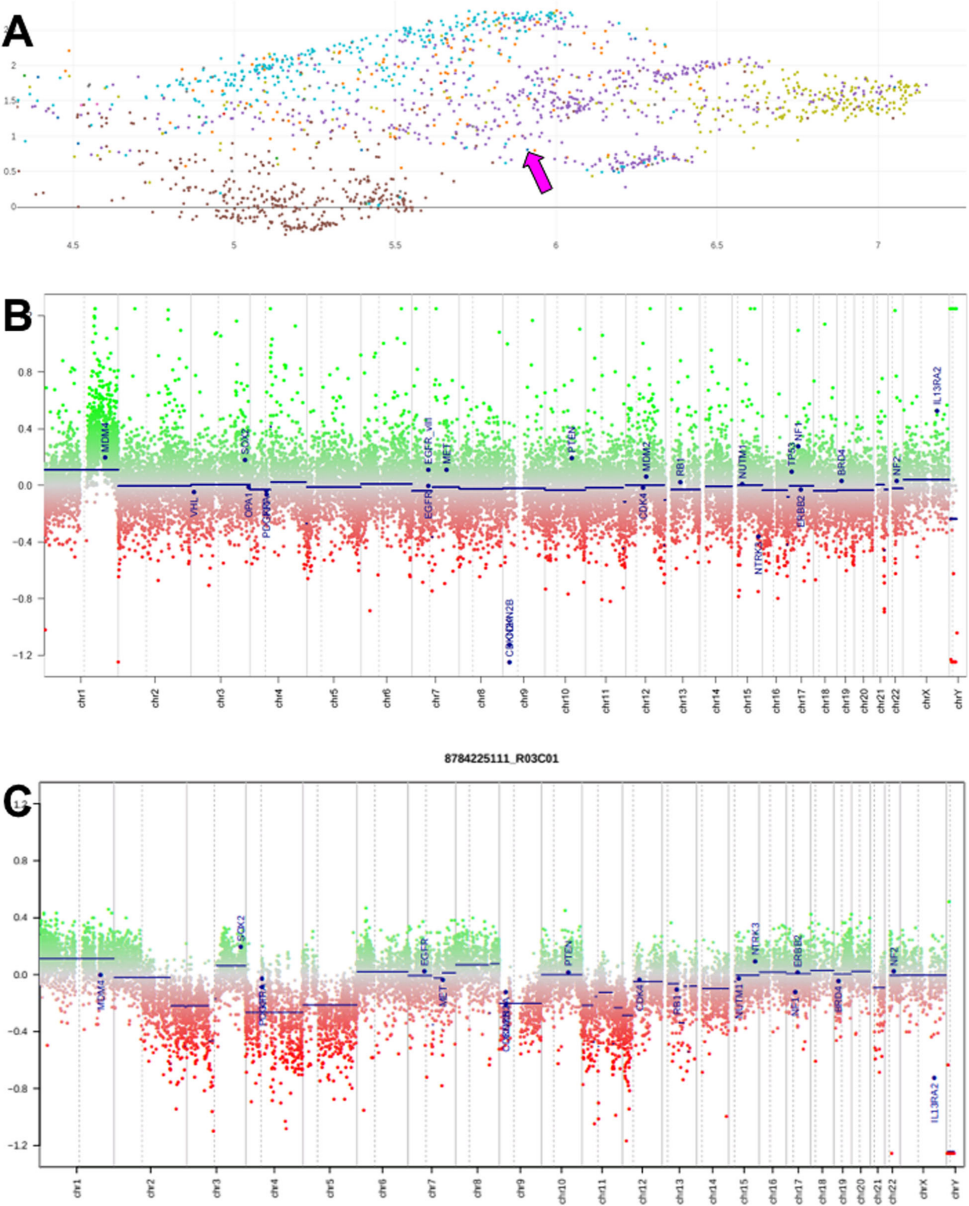
Microarray data analysis: **a** Region of interest within UMAP plot of the top 25′000 differentially methylated sites in the genome reveals that the current case (magenta arrow) does not fall into the centre of classical cervical squamous cell carcinomas (yellow) but overlaps with the methylation classes of squamous cell carcinomas of various origins (other colors); brown dots at bottom: bladder cancer. **b**, **c** Copy number profiles of tumour-derived DNA, calculated with R/conumee from methylation array data; **b** this case; **c** typical copy number profile of cervical squamous cell carcinoma (from TCGA reference cohort)

We performed molecular analyses of the primary tumour. The tumour was positive for PD-L1 (clone SP263, Ventana), as 30% of tumour infiltrating immune cells were PD-L1 positive (defined as the proportion of tumour area occupied by PD-L1 staining of any intensity in immune cells), whereas the tumour cells were PD-L1 negative. Unexpectedly, the tumour was negative for p16 by immunohistochemistry, so that an HPV-association appeared unlikely. A qPCR-based HPV test (Anyplex™ II HPV28 Detection, Seegene) on DNA from formalin-fixed, paraffin-embedded tissue was negative. To further characterize the neoplasm, we performed a genome-wide DNA methylation analysis (Infinium Methylation EPIC, Illumina) and compared the methylation pattern by a combined filtering and dimension reduction approach [12, 13] with reference data on various cancer types (The Cancer Genome Atlas, TCGA) including HPV-associated cervical squamous cell carcinomas, as specific HPV-associated structural aberrations and increased target-gene expression have been reported [14]. We observed the highest concordance with the methylation classes of squamous cell carcinomas, particularly of the cervix, head and neck, and oesophagus (Fig. 3a. Methylation array data were also analysed for copy number changes, revealing a relatively flat profile (Fig. 3b,c).

TMB was exceptionally high with 44 mutations/megabase (Oncomine™ Tumor Mutation Load Assay, Thermo Fisher Scientific), even though cut-off levels for non-HPV-related cervical cancers have yet to be defined. The TMB sequencing data were further screened for disease-related somatic variants. They revealed a pathogenic mutation in the *PIK3CA* gene and a likely pathogenic mutation in the *ERBB2* gene with allelic frequencies close to 40%, matching the estimated tumour proportion of 80%). Somatic mutations in both of these genes have been suggested to play a role in the pathogenesis of cervical squamous cell carcinoma [15].

Immunohistochemistry for DNA damage repair proteins (MLH1, MSH2, MSH6, PMS2) showed preserved expression of all examined proteins, consistent with a microsatellite stable (MSS) carcinoma.

## Discussion

We report on a patient with primary advanced cervical cancer with paraaortic lymph node metastases, which developed a complete and persistent remission under third-line therapy with nivolumab. Immunotherapy with checkpoint inhibitors is an emerging option for many types of solid cancers, including advanced cervical cancer for which data remain limited [6].

The PD-1/PD-L1 pathway is one of the most widely understood immune mechanisms involved in cancer, including in cervical carcinoma. PD-L1 expression has been reported in 95% of cervical intraepithelial neoplasms and 80% of squamous cell carcinomas while it was absent in normal cervical mucosa [1]. Persistent HPV infections are known to be involved in cervical carcinogenesis and to correlate with a significant PD-L1 up-regulation in tumour cells [16]. Checkmate-358 is a phase I/II trial investigating the response to nivolumab in HPV-associated advanced cervical (*n* = 19) as well as vaginal and vulvar (*n* = 5) cancers [17]. The median progression-free survival was 5.5 months, with a 6-month OS rate of 87.1%. In cervical cancer patients, a disease control rate of 68.4% and an ORR of 26.3% have been observed after one or more systemic therapies in recurrent or metastatic settings [17]. Pembrolizumab was evaluated in recurrent metastatic cervical cancer in the Keynote 028 phase Ib trial (*n* = 24) [18]. The Keynote 158 phase II trial (*n* = 98) showed an ORR of 17 and 12.2%, respectively [16, 17]. PD-L1 expression seems to be an important predictive biomarker in this setting. While the ORR increased up to 14.6% in PD-L1 positive cancers (> 80% of cases), no therapeutic response was seen in PD-L1 negative tumours [19]. Therefore, accelerated approval was granted for patients with advanced PD-L1 positive cervical cancer who progressed during or after chemotherapy [19]. Treatment with nivolumab is equal or superior to second or third-line chemotherapy based on the evidence from phase II trials.

In addition, methylation profiling might represent an independent modality to predict response to immune checkpoint inhibitors as recently demonstrated for lung cancer [10]. To facilitate data comparison with other cases we have included the raw methylation data as Additional file 1.

Particularly striking in comparison to the majority of (HPV-associated) cervical squamous cell carcinomas within the reference collection was the rather flat copy number profile of our case, hinting at a potential defect in DNA repair causing point mutations (not detectable with the methylation array) rather than being driven by a virus. It is, however, not entirely to be excluded that this unusual type of cancer described here evolved through infection with a rare, undetectable HPV genotype.

A strong association between TMB and response to immune checkpoint inhibitors has been reported for various cancer entities [7]. TMB and MSI-high also seem to correlate in several gynaecological cancers. However, in cervical cancer, where high TMB was seen in 6% of cases and MSI-high in 2.1% of cases, no significant correlation between both biomarkers was found [20].

Taken together, PD-L1 positivity of 30% and the high TMB (44 mutations/Mb) seem to be predictive biomarkers for response to immunotherapy and might explain the complete remission under nivolumab treatment.

As immunotherapy is used more and more frequently, it is imperative to gain insight into the management of IRAEs, which differ from classical chemotherapy side effects and might be life-threatening. The most common IRAEs affect the skin, mainly showing lichenoid reactions, eczema, vitiligo, and pruritus. Up to 30–40% of patients under nivolumab develop cutaneous reactions [21]. Cutaneous IRAEs are generally treated with topical or systemic corticosteroids, which mostly leads to their resolution within 6–12 weeks [11, 22]. If symptoms are steroid-refractory, immunosuppressive agents like TNF-alpha antagonists, azathioprine or mycophenolate mofetil may be tried [21]. In the case of severe reactions, interruption of immunotherapy should be considered [11].

Isolated vulvitis has not yet been described as an IRAE. Given the broad range of potential differential diagnosis, clinicians should be aware of the importance of biopsies in determining the underlying aetiology. Early referral to a gynaeco-oncologist is crucial to ensure appropriate management. The diagnosis of immune-related vulvitis is also important to avoid the evolution of an untreated vulvopathy [22]. Although in most cases, dermatological IRAEs are manageable and reversible, the malignant potential of untreated lesions is unknown and requires further investigation.

Patients with prior radiotherapy might be more likely to develop localised and/or more severe cutaneous IRAEs in irradiated skin regions. As in our patient, the vagina had been irradiated while the vulva was outside the irradiation field, a low dose radiotherapy effect on the vulvar skin cannot be excluded. In the literature, a case of nivolumab-induced Stevens-Johnson syndrome with striking enhancement at the site of radiation has been reported in a patient with metastatic squamous cell carcinoma of the oropharynx [23].

The evaluation of the impact of immunotherapy and IRAEs on the quality of life is an important aspect to consider. Clinical trials in patients with advanced solid tumours showed that treatment with nivolumab causes fewer and less severe adverse events in comparison to conventional chemotherapy [25]. Nevertheless, a vulvitis as reported in our case may impact on the quality of life, sexual wellbeing and compliance of patients. Early recognition and adequate management of IRAEs are therefore essential.

## Conclusions

Immunotherapy has emerged as a new option for patients with advanced cervical cancer.

We report the case of a patient with persistent complete response after third-line treatment with nivolumab for relapsed, chemotherapy-resistant cervical squamous cell carcinoma showing the promising potential of immunotherapy. Interestingly, the HPV-negative tumour was positive for PD-L1, had a very high TMB, and a comparably flat copy number profile.

To our knowledge, this is the first report of an immune-related vulvitis under immune checkpoint blockade. This IRAE was successfully treated with topical corticosteroids, which - by enabling nivolumab continuation - led to an excellent clinical outcome. Furthermore, we supplement our article with the tumour methylome dataset in order to provide the base for identification of comparable responders.

## Supplementary information

**Additional file 1.** IDAT methylation array data (Illumina Infinium EPIC) in zipped IDAT format (*_Grn.idat and *_Red.idat). These files are the output of the array scanner and can be used to calculate DNA methylation beta values as well as copy number profiles.

## Data Availability

The methylome data are included as an Additional file. All other datasets obtained during workup of this case are available from the corresponding author on reasonable request.

## Abbreviations

AUC: Area under the curve
CT: Computer tomography
HPV: Human papillomavirus
IRAE: Immune-related adverse event
MSI-high: High levels of microsatellite instability
ORR: Objective response rate
OS: Overall survival
PD-1: Programmed cell death protein 1
PD-L1: Programmed death-ligand 1
PET-CT: Positron emission tomography-CT
RR: Response rate
TGCA: The Cancer Genome Atlas
TMB: Tumour mutational burden
UMAP: Uniform Manifold Approximation and Projection for Dimension Reduction

## References

[1] Heong V, Ngoi N, Tan DSP (2017). Update on immune checkpoint inhibitors in gynecological cancers. J Gynecol Oncol.

[2] Tumeh PC, Harview CL, Yearley JH, Shintaku IP, Taylor EJM, Robert L (2014). PD-1 blockade induces responses by inhibiting adaptive immune resistance. Nature..

[3] Monk BJ, Sill MW, McMeekin DS, Cohn DE, Ramondetta LM, Boardman CH (2009). Phase III trial of four cisplatin-containing doublet combinations in stage IVB, recurrent, or persistent cervical carcinoma: a gynecologic oncology group study. J Clin Oncol Off J Am Soc Clin Oncol.

[4] Minion LE, Tewari KS (2018). Cervical cancer - state of the science: from angiogenesis blockade to checkpoint inhibition. Gynecol Oncol.

[5] Tewari Krishnansu S., Sill Michael W., Long Harry J., Penson Richard T., Huang Helen, Ramondetta Lois M., Landrum Lisa M., Oaknin Ana, Reid Thomas J., Leitao Mario M., Michael Helen E., Monk Bradley J. (2014). Improved Survival with Bevacizumab in Advanced Cervical Cancer. New England Journal of Medicine.

[6] Liu Y, Wu L, Tong R, Yang F, Yin L, Li M (2019). PD-1/PD-L1 inhibitors in cervical Cancer. Front Pharmacol.

[7] Goodman AM, Kato S, Bazhenova L, Patel SP, Frampton GM, Miller V (2017). Tumor mutational burden as an independent predictor of response to immunotherapy in diverse cancers. Mol Cancer Ther.

[8] Chan TA, Yarchoan M, Jaffee E, Swanton C, Quezada SA, Stenzinger A (2019). Development of tumor mutation burden as an immunotherapy biomarker: utility for the oncology clinic. Ann Oncol Off J Eur Soc Med Oncol.

[9] Le DT, Durham JN, Smith KN, Wang H, Bartlett BR, Aulakh LK (2017). Mismatch repair deficiency predicts response of solid tumors to PD-1 blockade. Science.

[10] Duruisseaux M, Martínez-Cardús A, Calleja-Cervantes ME, Moran S (2018). Castro de Moura M, Davalos V, et al. epigenetic prediction of response to anti-PD-1 treatment in non-small-cell lung cancer: a multicentre, retrospective analysis. Lancet Respir Med.

[11] Haanen JBAG, Carbonnel F, Robert C, Kerr KM, Peters S, Larkin J (2017). Management of toxicities from immunotherapy: ESMO Clinical Practice Guidelines for diagnosis, treatment and follow-up. Ann Oncol Off J Eur Soc Med Oncol.

[12] Capper D, Jones DTW, Sill M, Hovestadt V, Schrimpf D, Sturm D (2018). DNA methylation-based classification of central nervous system tumours. Nature..

[13] McInnes L, Healy J, Melville J. UMAP: Uniform Manifold Approximation and Projection for Dimension Reduction. ArXiv. 2018:180203426 Available from: http://arxiv.org/abs/1802.03426. Cs Stat [Internet] [cited 2019 Jun 13].

[14] The Cancer Genome Atlas Research Network (2017). Integrated genomic and molecular characterization of cervical cancer. Nature..

[15] Ojesina AI, Lichtenstein L, Freeman SS, Pedamallu CS, Imaz-Rosshandler I, Pugh TJ (2014). Landscape of genomic alterations in cervical carcinomas. Nature..

[16] Mezache L, Paniccia B, Nyinawabera A, Nuovo GJ (2015). Enhanced expression of PD L1 in cervical intraepithelial neoplasia and cervical cancers. Mod Pathol Off J U S Can Acad Pathol Inc.

[17] Hollebecque A, Meyer T, Moore KN, Machiels J-PH, De Greve J, López-Picazo JM (2017). An open-label, multicohort, phase I/II study of nivolumab in patients with virus-associated tumors (CheckMate 358): Efficacy and safety in recurrent or metastatic (R/M) cervical, vaginal, and vulvar cancers. J Clin Oncol.

[18] Frenel Jean-Sebastien, Le Tourneau Christophe, O’Neil Bert, Ott Patrick A., Piha-Paul Sarina A., Gomez-Roca Carlos, van Brummelen Emilie M.J., Rugo Hope S., Thomas Shari, Saraf Sanatan, Rangwala Reshma, Varga Andrea (2017). Safety and Efficacy of Pembrolizumab in Advanced, Programmed Death Ligand 1–Positive Cervical Cancer: Results From the Phase Ib KEYNOTE-028 Trial. Journal of Clinical Oncology.

[19] Chung Hyun Cheol, Ros Willeke, Delord Jean-Pierre, Perets Ruth, Italiano Antoine, Shapira-Frommer Ronnie, Manzuk Lyudmila, Piha-Paul Sarina A., Xu Lei, Zeigenfuss Susan, Pruitt Scott K., Leary Alexandra (2019). Efficacy and Safety of Pembrolizumab in Previously Treated Advanced Cervical Cancer: Results From the Phase II KEYNOTE-158 Study. Journal of Clinical Oncology.

[20] Winer I, Jones NL, Xiu J, Ellerbrock A, Brown J, Herzog T (2018). Mutational burden, tumor immune checkpoint expression, and microsatellite instability in gynecologic malignancies: implications for immune therapy. Gynecol Oncol.

[21] Michot JM, Bigenwald C, Champiat S, Collins M, Carbonnel F, Postel-Vinay S (2016). Immune-related adverse events with immune checkpoint blockade: a comprehensive review. Eur J Cancer.

[22] Halonen P, Jakobsson M, Heikinheimo O, Riska A, Gissler M, Pukkala E (2017). Lichen sclerosus and risk of cancer. Int J Cancer.

[23] Shah KM, Rancour EA, Al-Omari A, Rahnama-Moghadam S. Striking enhancement at the site of radiation for nivolumab-induced Stevens-Johnson syndrome. Dermatol Online J. 2018;24(6):7 30142712

[24] Shah KM, Rancour EA, Al-Omari A, Rahnama-Moghadam S. Striking enhancement at the site of radiation for nivolumab-induced Stevens-Johnson syndrome. Dermatol Online J. 2018 Jun 15;24(6):7. 30142712

[25] Tykodi SS, Schadendorf D, Cella D, Reck M, Harrington K, Wagner S (2018). Patient-reported outcomes with nivolumab in advanced solid cancers. Cancer Treat Rev.

